# Prevalence of Musculoskeletal Pain in Construction Workers in Saudi Arabia

**DOI:** 10.1155/2015/529873

**Published:** 2015-02-25

**Authors:** Ahmad Alghadir, Shahnawaz Anwer

**Affiliations:** ^1^Department of Rehabilitation Sciences, College of Applied Medical Sciences, King Saud University, Riyadh, Saudi Arabia; ^2^Padmashree Dr. D. Y. Patil College of Physiotherapy, Dr. D. Y. Patil Vidyapeeth, Pune, India

## Abstract

The objective of this study was to find out the prevalence, characteristics, and distribution of musculoskeletal pain among construction workers in Saudi Arabia. A questionnaire about musculoskeletal pain in different parts of the body was completed by 165 construction workers from the construction industries in Dammam and Riyadh cities. The descriptive data were analyzed using chi-square test. The level of statistical significance was set at *P* < 0.05. Eighty (48.5%) of the responding workers had pain in neck, shoulders, lower back, hand, knee, or ankle. The majority of respondents had low back pain (50%) followed by knee pain (20%). The average intensity of pain at all sites during activity and rest was 6.65 and 3.59, respectively. Thirty-four (42.5%) respondents had dull aching pain and 24 (30%) had cramping pain. There was an association between years of experience, duration of break during work, and use of protective equipment with the prevalence of musculoskeletal pain in construction workers (*P* < 0.05). Most of the workers complaining of pain got medical treatment (62.5%) and only 25% received physical therapy. It can be concluded from this study that the prevalence of musculoskeletal pain among construction workers in Saudi Arabia is high.

## 1. Introduction

Musculoskeletal pain (MSP) is one of the prevailing occupational health problems, and workers in the construction industry have potential risk of MSP. Worldwide, the prevalence of musculoskeletal symptoms involving one or more body regions is higher in construction workers [1]. The load of physical work associated with awkward prolong working postures and manual handling of materials by the construction workers can cause various musculoskeletal pains and disorders [2].

Musculoskeletal disorders (MSDs) cause decreased health and work ability, thereby increasing the costs of absenteeism due to less productivity at work [3]. In the US a nationwide health interview survey showed that construction workers are the highest risk group for work-related low-back pain [4]. In a British study, the 1-year cumulative incidence of low-back pain was 40% for construction workers as compared with 28% for managers [5].

Work-related musculoskeletal symptoms are the commonest cause of occupational disability among construction workers [6]. Most studies on work-related musculoskeletal symptoms were limited to office, service, or manufacturing industries. However, the construction industry is considered as one of the most hazardous industries for work-related musculoskeletal symptoms among 10 most frequently reported industries [7, 8].

One Indian study reported that four in five construction workers had symptoms of MSDs [9]. Joshi et al. have reported 59.4% prevalence of MSDs in their study on workers and have suggested that the high prevalence of MSDs in workers needs urgent attention from the health and labor sectors [10]. Aghilinejad et al. [11] reported high rate of musculoskeletal complaints in Iranian aluminum industries. Another study reported potential risk of MSDs in brick field workers in India [12]. Till date no data on work-related musculoskeletal pain is recorded for construction workers among the Saudi population. Our survey aimed to find out prevalence, characteristics, and distribution of musculoskeletal pain among construction workers in Saudi Arabia.

## 2. Methods

A descriptive cross-sectional study was used in this research. A sample consisting of 165 male construction workers belonging to 9 main jobs from the construction industries in Dammam and Riyadh cities in the age group of 18–60 years was selected and invited to participate in this survey. Participation was completely voluntary and there were no personal identifiers. This study was approved by the institutional ethical committee, King Saud University, and written consent of each subject was taken for their voluntary participation. A questionnaire was developed and validated. The questionnaire included demographic data, personal data, work related history, presence or absence of any musculoskeletal pain in the last 12 months, affected body parts, the intensity of pain on a numerical rating scale (NRS) [13], and any treatment taken. One evaluator conducted face-to-face surveys with structured interviews with construction workers.

### 2.1. Statistical Analysis

Initial database entry was performed using Excel 2007 (Microsoft Corp, Redmond, WA, USA). Statistical analysis was done using SPSS 22.0 Software (SPSS, IBM Inc.). Descriptive statistics, including proportions, means, medians, and standard deviations, were calculated for age, height, weight, BMI, marital status, job, habit of smoking, educational status, working year, working hours, usual working posture, presence of musculoskeletal pain, affected body part, the intensity of pain, type of pain, and type of treatment taken. The differences in prevalence were analyzed using chi-square tests at statistical significance level of 0.05.

## 3. Results

Table 1 details the participation characteristics. The sample consisted of 42 (25.5%) manual laborers, 10 (6.1%) carpenters, 11 (6.7%) bricklayers, 11 (6.7%) painters, 21 (12.7%) electricians, 28 (17%) plumbers, 10 (6.1%) interior finish worker, 7 (4.2%) scaffolders, 15 (9.1%) crane operators, and 10 (6.1%) others. A hundred and thirty (78.8%) respondents were married and more than 90% of respondents were literate. Sixty-three (38.2%) respondents had a history of smoking.

Table 2 details the prevalence of musculoskeletal pain among respondents. Of the 165 construction workers, 80 (48.5%) reported musculoskeletal pain. The majority of respondents had pain in the low back (50%) followed by knee (20%), neck (8%), shoulder (8%), ankle/foot (6.3%), elbow (2.5%), hand (2.5%), or upper back (1.6%). More than 70% of respondents who reported musculoskeletal pain were in the age group of 30–50 years. The majority of respondents who reported pain were married, overweight, and obese. 45% of the respondents who reported pain had a history of smoking. Respondents having less than 5 years of working experience reported more pain. Respondents who worked more than 8 hours/day reported more pain. Additionally, respondents who took more than 20 minutes break during work reported less pain. Also 55% of respondents who reported pain were using protective equipment.

Table 3 details the characteristics and consequences of musculoskeletal pain in construction workers. The majority of respondents had low back pain (50%) followed by knee pain (20%). The average intensity of pain during activity and rest was 6.65 and 3.59, respectively. Most of the respondents had periodic pain (50%) followed by regular pain (28.8%). The duration of pain persisted for 2–4 days in most of the workers (47.5%). Thirty-four (42.5%) respondents had dull aching pain and 24 (30%) had cramping pain. Thirty-eight (47.5%) respondents who reported pain took more than 15-day sick leave in last 12 months. However, 45% of the respondents who reported pain did not take any sick leave. The analysis of the questions related to the usual position of work showed that the most frequently used postures were standing and sitting postures (65% and 37%, resp.). Fifty (62.5%) workers complaining of pain got medical treatment; only 25% got physical therapy.

## 4. Discussion 

This survey aimed to find out the prevalence, characteristics, and distribution of musculoskeletal pain among construction workers in Saudi Arabia. The 12-month prevalence of musculoskeletal pain in construction workers was high (48.5%). Our findings are consistent with the previous cross-sectional questionnaire based study that reported a similar prevalence of musculoskeletal complaints in construction workers [14–17]. Merlino et al. [18] and Bodhare et al. [9] reported very high prevalence of musculoskeletal disorders with 76.8% and 77%, respectively. In contrast, Guo et al. [7] reported a little lower prevalence of musculoskeletal disorders of 37%.

In the present study, the most common site of pain was lower back which is similar to previous findings among construction workers [4, 14, 15, 17–23]. The prevalence of knee pain in this study was high (20%) after the lower back. This finding is in agreement with those of Merlino et al. [18] who reported high prevalence of knee pain (38.4%) after low back pain in their samples of young construction workers.

In this study, we investigated the average intensity of pain during activity and rest among construction workers. The average intensity of pain during activity was more than 7 on NRS in 56.3% of construction workers. The average intensity of pain at rest was 4–6 on NRS in 51.3% of them. The intensity of pain is useful to get an insight into the severity of the musculoskeletal symptoms. The high intensity of pain during activity and at rest in the present study suggests that construction workers are at risk of severe musculoskeletal disorders. However, this important aspect was not investigated previously.

The average duration of pain was 2–4 days in 47.5% construction workers, for which they took more than 15 days sick leave over last 12 months. In the present study, the most prevalent type of pain was dull aching (42.5%) followed by cramping (30%). The type of pain can give us a clue to determine which structures are affected. Also this aspect of pain was not investigated previously.

The present study found an association between years of experience, duration of break during work, and use of protective equipment with the prevalence of musculoskeletal pain in construction workers. The workers who had worked more than 5 years had an increased prevalence of MSP. On the other hand, those who took longer break durations (>20 minutes) and used protective equipment during work reported less MSP. In a previous study, Kaminskas and Antanaitis [16] reported an association between musculoskeletal pain and years in the construction industry. The prevalence of musculoskeletal pain was 33% in workers who work less than 5 years in the industry. The prevalence increases to 40% when working years are 6–10 years. The prevalence further increases to 84% when the working years increase to 30 years. However, in the present study we categorized working years into two groups: less than 5 years and more than 5 years. No study previously investigated the association of MSP with duration of the break and usage of protective equipment during work. However, these two could be important factors to prevent the occurrence of work related MSP.

There are several limitations to the present study. The study was limited to only male respondents. The cause and effect cannot be established as this study was a cross-sectional study. The different circumstances of questionnaire administration such as self-reported or interview-based should be considered and the comparison of results should always be made with some caution. The questionnaire administered through interviews, as in this study, instead of self-reported, can ensure greater validity of the answers.

## 5. Conclusions

It can be concluded that the prevalence of musculoskeletal pain among construction workers in Saudi Arabia is high. The majority of respondent had low back pain followed by knee pain. The high intensity of pain during activity and at rest in the present study suggests that construction workers are at risk of severe musculoskeletal disorders. The most prevalent type of pain was dull aching followed by cramping. There was an association of years of experience, duration of break during work, and use of protective equipment with the prevalence of musculoskeletal pain in construction workers.

## Figures and Tables

**Table 1:** Participants characteristics.

Age, years	
Mean (SD)	34.82 (8.33)
Range	20–55
Height, meter (m)	
Mean (SD)	1.66 (0.06)
Range	1.50–1.89
Weight, kg	
Mean (SD)	72.70 (12.75)
Range	43–108
BMI, kg/m^2^	
Mean (SD)	26.13 (3.76)
Range	17.01–36.51
Marital status, number (%)	
Married	130 (78.8)
Single	35 (21.2)
Educational status, number (%)	
Illiterate	15 (9.1)
Primary	55 (33.3)
Secondary	75 (45.5)
Graduation	20 (12.1)
Smoking, number (%)	
Yes	63 (38.2)
No	102 (61.8)
<20 cigarettes/day	44 (69.9)
>20 cigarettes/day	19 (30.1)
Type of work, number (%)	
Manual laborer	42 (25.5)
Plumber	28 (17)
Electrician	21 (12.7)
Crane operators	15 (9.1)
Bricklayers	11 (6.7)
Painter	11 (6.7)
Carpenter	10 (6.1)
Interior finish worker	10 (6.1)
Scaffolders	7 (4.2)
Others	10 (6.1)

**Table 2:** Prevalence of musculoskeletal pain among respondents.

Variables	Yes (%)	No (%)	Chi-square test
Musculoskeletal pain	80 (48.5)	85 (51.5)	*P* value

Age, years			0.137
20–29	20 (25)	32 (37.6)	
30–39	31 (38.7)	32 (37.6)	
40–49	26 (32.6)	16 (18.8)	
50–59	3 (3.7)	5 (5.8)	
Marital status			0.258
Married	66 (82.5)	64 (75.2)	
Single	14 (17.5)	21 (24.8)	
Educational status			0.206
Illiterate	9 (11.2)	6 (7.2)	
Primary	22 (27.5)	33 (38.8)	
Secondary	36 (45)	39 (45.8)	
Graduation	13 (16.3)	7 (8.2)	
BMI			0.174
Underweight	2 (2.5)	0 (0)	
Normal weight	26 (32.5)	38 (44.7)	
Over weight	35 (43.7)	35 (41.1)	
Obese	17 (21.2)	12 (14.1)	
Smoking			0.080
Yes	36 (45)	27 (31.8)	
No	44 (55)	58 (68.2)	
Type of work			0.565
Manual laborer	20 (25)	22 (25.8)	
Plumber	16 (20)	12 (14.1)	
Electrician	8 (10)	13 (15.3)	
Crane operators	9 (11.2)	6 (7)	
Bricklayers	7 (8.7)	4 (4.7)	
Painter	7 (8.7)	4 (4.7)	
Carpenter	3 (3.7)	7 (8.2)	
Interior finish worker	3 (3.7)	7 (8.2)	
Scaffolders	3 (3.7)	4 (4.7)	
Others	4 (5)	6 (7)	
Years of experience			0.011^*^
>5 years	39 (48.8)	25 (29.4)	
<5 years	41 (51.2)	60 (70.6)	
Working hours/day			0.603
>8 hour/day	41 (51.2)	47 (55.3)	
<8 hour/day	39 (48.8)	38 (44.7)	
Duration of break during work			0.002^*^
>20 minutes	30 (37.5)	16 (18.9)	
<20 minutes	50 (62.5)	69 (81.1)	
Use of protective equipment			0.001^*^
Yes	44 (55)	67 (78.9)	
No	36 (45)	18 (21.1)	

^*^ *P* < 0.05.

**Table 3:** Characteristics and consequences of musculoskeletal pain in construction workers.

	Musculoskeletal pain sufferers, *N* = 80
Location of pain, number (%)	
Neck	7 (8.8)
Shoulder	7 (8.8)
Upper back	1 (1.3)
Elbow	2 (2.5)
Hand	2 (2.5)
Low back	40 (50)
Knee	16 (20)
Ankle/foot	5 (6.3)
Average intensity of pain during activity, NRS (0–10), number (%)	
1–3	2 (2.5)
4–6	33 (41.3)
7–10	45 (56.3)
Average intensity of pain at rest, NRS (0–10), number (%)	
1–3	38 (47.5)
4–6	41 (51.3)
7–10	1 (1.3)
Frequency of pain, number (%)	
Occasional	40 (50)
Often	23 (28.8)
Sometimes	11 (13.8)
Always	6 (7.5)
Duration of pain, number (%)	
<2 hours	20 (25)
>2 hours	6 (7.5)
2–4 days	38 (47.5)
>1 week	16 (20)
Type of pain, number (%)	
Cramping	24 (30)
Dull aching	34 (42.5)
Shooting	16 (20)
Burning	6 (7.5)
Sick leave, number (%)	
Nil	36 (45)
<15 days	6 (7.5)
>15 days	38 (47.5)
Any kind of treatment taken, number (%)	
Medical	50 (62.5)
Physiotherapy	20 (25)
Others	10 (12.5)
